# A Clinical Text-Book of Medical Diagnosis

**Published:** 1899-12

**Authors:** 


**A Clinical Text-Book of Medical Diagnosis.** By Oswald Vierordt, M.D. Authorized translation by Francis H. Stuart, M.D. Fourth American Edition, from the Fifth German. Pp. 603. London: The Rebman Publishing Co. (Ltd.). Philadelphia: W. B. Saunders. 1898.

A veritable mine of information on all points in medical diagnosis, this book is one of the best which has yet been written on the subject. To become an accomplished diagnostician is the aim of every physician: the particular purpose of this work is to furnish the physician with the material by which he may attain his object. "The foundation of a correct diagnosis must rest upon a careful examination of the individual organs, and then a study of the whole organism, the totality of the picture of the disease." We are somewhat surprised to find that the centrifuge is not mentioned for the examination of blood.

It is impossible within any ordinary limits to give an adequate idea of the merits of this volume, which should be not only in the hands of every clinical clerk, but should also be the cherished and oft-used possession of every one upon whom rests the responsibility of medically treating sick persons.

